# Mechanism of Peitu Shengjin Formula Shenlingbaizhu Powder in Treating Bronchial Asthma and Allergic Colitis through Different Diseases with Simultaneous Treatment Based on Network Pharmacology and Molecular Docking

**DOI:** 10.1155/2022/4687788

**Published:** 2022-05-09

**Authors:** Liying Zeng, Shaodan Sun, Peiwen Chen, Qina Ye, Xiaoling Lin, Hongjun Wan, Yawen Cai, Xiaogang Chen

**Affiliations:** ^1^The First Clinical College, Guangzhou University of Chinese Medicine, Guangzhou 510405, Guangdong, China; ^2^The Second Affiliated Hospital, Guangzhou University of Chinese Medicine, Guangzhou 510120, Guangdong, China; ^3^Guangzhou Women and Children Medical Center, Guangzhou 510623, Guangdong, China; ^4^The First Affiliated Hospital, Guangzhou University of Chinese Medicine, Guangzhou 510405, Guangdong, China

## Abstract

**Background:**

Shenlingbaizhu powder (SLBZP), one of the classic Earth-cultivating and gold-generating prescriptions of traditional Chinese medicine, is widely used to treat various diseases. However, the pharmacological mechanisms of SLBZP on bronchial asthma (BA) and allergic colitis (AC) remain to be elucidated.

**Methods:**

Network pharmacology and molecular docking technology were used to explore the potential mechanism of SLBZP in treating BA and AC with the simultaneous treatment of different diseases. The potential active compounds of SLBZP and their corresponding targets were obtained from BATMAN-TCM, ETCM, SymMap TCM@TAIWAN, and TCMSP databases. BA and AC disease targets were collected through DisGeNET, TTD, GeneCards, PharmGKB, OMIM, NCBI, The Human Phenotype Ontology, and DrugBank databases. Common targets for drugs and diseases were screened by using the bioinformatics and evolutionary genomics platform. The analyses and visualizations of Gene Ontology (GO) function and Kyoto Encyclopedia of Genes and Genomes (KEGG) pathway enrichment of common targets were carried out by *R* software. The key targets were screened by using the plug-in “cytoHubba” of Cytoscape software, and the “active compound-key target” network was constructed. Molecular docking analysis was performed using AutoDock software. The miRTarBase database was used to predict microRNAs (miRNAs) targeting key targets, and the key target-miRNA network was constructed.

**Result:**

Through screening, 246 active compounds and 281 corresponding targets were obtained. Common targets were mainly enriched in 2933 biological processes and 182 signal pathways to play the role of treating BA and AC. There were 131 active compounds related to key targets. The results of molecular docking showed that the important active compounds in SLBZP had good binding ability with the key targets. The key target-miRNA network showed that 94 miRNAs were predicted.

**Conclusion:**

SLBZP has played the role of treating different diseases with the same treatment on BA and AC through the characteristics of multicompound, multitarget, and multipathway of traditional Chinese medicine, which provides a theoretical basis for explaining the mechanism and clinical application of SLBZP treating different diseases with the same treatment in BA and AC.

## 1. Introduction

Asthma generally refers to bronchial asthma (BA). BA, one of the most common chronic noncommunicable diseases in children and adults, is characterized by variable respiratory symptoms and variable airflow limitation, which is the result of complex gene-environment interactions, and is heterogeneous in clinical manifestations and the type and intensity of airway inflammation and remodeling [1]. The goal of BA treatment is to achieve good asthma control, that is, to minimize the burden of symptoms and the risk of deterioration [2]. However, asthma attacks and hospitalizations are frequent, and the mortality rate remains high. Strategies need to be developed to change the natural history of BA and prevent serious deterioration and the decline of lung function [1]. Allergic colitis (AC), an inflammatory disease, is characterized by the infiltration of eosinophils into the colon wall and the presence of red blood in the stool of healthy breast-fed or formula-fed infants, which usually develops in the first few weeks or months of life and can be a benign and/or severe disease in infant gastrointestinal diseases [3–4]. To date, the most effective interventions are preventive methods, especially feeding strategies, to reduce the incidence of disease while establishing adequate growth and progression to enteral feeding [5]. However, their pathogenesis has not yet been fully clarified with some allergens unclear or unavoidable, and modern medicine lacks ideal preventive and therapeutic methods [6]. At present, modern medicine adopts allergen avoidance, desensitization, and symptomatic treatment, but some antihistamines and antileukotrienes need to be taken for a long time, which brings certain economic burden and psychological impact to patients and cannot completely cure allergic diseases with some deficiencies, such as side effects of drugs and easy recurrence after withdrawal [7–9]. In recent years, treating allergic diseases with traditional Chinese medicine has been more and more widely used in clinical practice with various methods, remarkable effects, less adverse reactions in long-term application, and good compliance, which is convenient for clinical promotion [10, 11].

Shelingbaizhu powder (SLBZP), from the Prescriptions *of Peaceful Benevolent Dispensary* and composed of 10 Chinese medicines including renshen (*Panax ginseng* C. A. Mey.), fuling (*Poria cocos* (Schw.) Wolf.), baizhu (Atractylodes macrocephala Koidz.), baibiandou (Lablab Semen Album), shanyao (Rhizoma Dioscoreae), lianzi (Semen Nelumbinis), yiyiren (Coicis Semen), sharen (*Amomum aurantiacum* H. T. Tsai Et S. W. Zhao), jiegeng (*Platycodon grandiforus*), and gancao (licorice), has the effects of replenishing qi, strengthening spleen, excreting dampness, and stopping diarrhea [12]. Previous studies have shown that SLBZP can regulate intestinal water metabolism and intestinal flora, inhibit inflammatory response, repair intestinal mucosal barrier, and enhance colonic motility, which is widely used in the clinical treatment of ulcerative colitis, chronic diarrhea, chronic obstructive pulmonary disease, bronchial asthma, diabetes, eczema, allergic rhinitis, etc. [13, 14].

**Figure 1:**
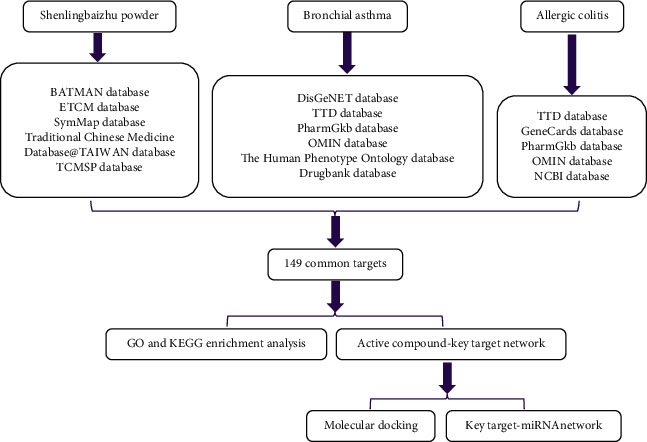
Workflow for exploring the mechanisms of Shenlingbaizhu powder in treating bronchial asthma and allergic colitis with simultaneous treatment of different diseases.

Network pharmacology, targeting biological networks, analyzes the connections between drugs, targets, and diseases in these networks. A comprehensive and systematic research on network pharmacology conforms to a holistic view, which is the main characteristic of many traditional medicines. Studies have shown that many traditional medicines exhibit synergistic effects by acting on multiple targets and pathways at different levels through network pharmacology [15]. This method effectively bridges the gap between modern medicine and traditional medicine and greatly promotes the research on the synergy of traditional medicine. Different diseases with simultaneous treatment means that the same pathogenesis appears in the occurrence and development of different diseases, and the same treatment can be adopted. SLBZP reinforces Earth to generate metal for treating BA and AC, which is in line with the concept of different diseases with simultaneous treatment. This study comprehensively analyzed and explored the mechanism of SLBZP in treating BA and AC with simultaneous treatment of different diseases from compounds, targets, pathways, biological processes, etc., by network pharmacology and molecular docking, which conforms to the overall function of traditional Chinese medicine theory and provides theoretical bases for clarifying the action mechanism of SLBZP on BA and AC and promoting its clinical application (Figure 1).

## 2. Materials and Methods

### 2.1. Screening Compounds and Targets of SLBZP

The active compounds of SLBZP were separately obtained from these databases: BATMAN-TCM (http://bionet.ncpsb.org.cn/batman-tcm/index.php/Home/Index/index) [16], ETCM (http://www.tcmip.cn/ETCM/index.php/Home/Index/) [17], SymMap (http://www.symmap.org/) [18] and Traditional Chinese Medicine Database@TAIWAN (http://tcm.cmu.edu.tw/review.php?menuid=3) [19]. Then, the active compounds that had good oral bioavailability (OB) and drug similarity (DL) and their targets of SLBZP were screened out under the conditions of OB ≥ 30% and DL ≥ 0.18 by entering the above obtained active compounds into Traditional Chinese Medicine Systems Pharmacology Database and Analysis Platform (TCMSP, http://lsp.nwu.edu.cn/tcmsp.php) [20]. Meanwhile, the active compounds and their targets of SLBZP from the TCMSP database were also obtained with OB ≥ 30% and DL ≥ 0.18. Next, all these obtained active compounds were synthesized to remove duplications. The full names of the targets screened by TCMSP were input into the DrugBank database (https://www.drugbank.ca/) [21] and UniProt database (https://www.uniprot.org/?tdsourcetag=s_pcqq_aiomsg) [22] to get the gene symbol and UniProt ID, which were all standardized and normalized to ensure accuracy.

### 2.2. Screening Targets of BA and AC

The target genes related to BA were obtained with the keyword “bronchial asthma” and the species set as “Homo sapiens” from these 8 databases: DisGeNET (http://www.disgenet.org/web/DisGeNET/menu/search) [23], TTD (https://db.idrblab.org/ttd/) [24], GeneCards (https://www.genecards.org) [25], PharmGKB (https://www.pharmgkb.org/) [26], OMIM (https://omim.org/) [27], NCBI (https://www.ncbi.nlm.nih.gov/gene) [28], The Human Phenotype Ontology (https://hpo.jax.org/app/) [29], and DrugBank. The target genes related to AC were obtained with the keyword “allergic colitis” and the species set as “Homo sapiens” from these 5 databases: TTD, GeneCards, PharmGKB, OMIM, and NCBI. The obtained data were combined separately, and then the duplications were removed. The full name of the last screened target genes were input into the DrugBank database and UniProt database to get the gene symbol and UniProt ID, which were also all standardized and normalized to ensure accuracy.

### 2.3. Screening of Common Targets

The targets related to active compounds, BA, and AC were matched and mapped by using the bioinformatics and evolutionary genomics platform (http://bioinformatics.psb.ugent.be/webtools/Venn/). At the same time, a Venn diagram was drawn to obtain the common targets of the active compounds of SLBZP for treating BA and AC.

### 2.4. GO and KEGG Enrichment Analysis of Common Targets

The enrichment analysis and visualization of Gene Ontology (GO) function and Kyoto Encyclopedia of Genes and Genomes (KEGG) pathways were carried out for the common targets of SLBZP in treating BA and AC with the species set as “Homo sapiens” and the threshold set as *P* *<* *0.05* by the “ggplot2”, “enrichplot”, “clusterprofiler” [30], and “ggpubr” packages of *R* software (version 3.6.1).

### 2.5. Construction of Active Compound-Key Target Network

The obtained common targets were imported into Cytoscape software (version 3.8.0; http://www.cytoscape.org) [31], and the “cytoHubba” plug-in was used to screen out the key targets. Then, an active compound-key target network was constructed by Cytoscape software, of which the network topology analysis was carried out by “Network Analysis” in the tool. The network showed the connection between the active compounds and key targets, and the molecular mechanism of SLBZP in treating BA and AC was explored on this basis.

### 2.6. Molecular Docking Verification

According to the above analysis results, the key target proteins and the important active compounds were molecularly docked. The protein structures of the targets were obtained from the RCSB PDB database (https://www.rcsb.org/) [32]. The 2D structures of the active compounds were obtained from the PubChem database (https://pubchem.ncbi.nlm.nih.gov/) [33] and were optimized to save as 3D structures with Chem3D software. AutoDockTools and AutoDockVina software were used for molecular structure processing and molecular docking. PyMOL and Discovery Studio were used to visualize the docking results.

### 2.7. Construction of Key Target-microRNA (miRNA) Network

The miRTarBase database (https://mirtarbase.cuhk.edu.cn/%7EmiRTarBase/miRTarBase_2019/php/index.php) is used to predict upstream miRNAs targeting key targets [34]. The collected miRNA-mRNA interactions have been verified by different types of experiments including report analyses in miRTarBase, western blot, qPCR, microarray, and next-generation sequencing experiments. In order to make predictions more reliable and accurate, only miRNAs that may interact with the targets were obtained through reporter gene analyses. After selecting “By Target Gene” and the species as “Human”, key targets were entered to predict miRNAs. Then, the key targets and their corresponding predicted miRNAs were organized into an Excel file that was imported into Cytoscape software. Finally, the network of the predicted miRNAs and key targets were constructed by Cytoscape software.

## 3. Results

### 3.1. Acquirement of Active Compounds of SLBZP

Preliminarily, a total of 335 active compounds were acquired from the BATMAN-TCM database; a total of 443 active compounds were acquired from the ETCM database; a total of 1182 active compounds were acquired from the SymMap database; a total of 352 active compounds were acquired from the Traditional Chinese Medicine Database@TAIWAN database; and a total of 171 active compounds were acquired from the TCMSP database. At last, 217 eligible unique active compounds of SLBZP in total were retrieved from the TCMSP database under the conditions of OB ≥ 30% and DL ≥ 0.18, which are all shown in Table 1.

### 3.2. Collection of BA and AC Disease Targets

4795 BA-related target genes were collected based on DisGeNET, TTD, GeneCards, PharmGKB, OMIM, NCBI, The Human Phenotype Ontology, and DrugBank databases. Duplicate targets were excavated and deleted, and 3388 BA disease action targets in total were collected. 1828 AC-related target genes were collected based on TTD, GeneCards, PharmGKB, OMIM, and NCBI databases. And 1640 AC disease action targets in total were collected by mining and deleting duplicate targets. The obtained target information was standardized for gene symbol and UniProt ID.

### 3.3. Acquirement of Targets of Active Compounds of SLBZP for Treating BA and AC

**Figure 2:**
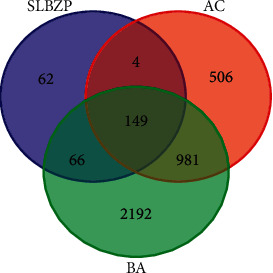
Targets matching among SLBZP, BA, and AC.

After searching the above-mentioned qualified potential active compounds of SLBZP in the TCMSP database, and removing the repeated targets, 281 targets of active compounds of SLBZP were obtained. The bioinformatics and evolutionary genomics platform was used to match the potential targets of drugs with disease targets, and a Venn diagram was drawn (Figure 2). 149 common targets were obtained (Table 2).

### 3.4. GO and KEGG Pathway Enrichment Analysis

**Figure 3:**
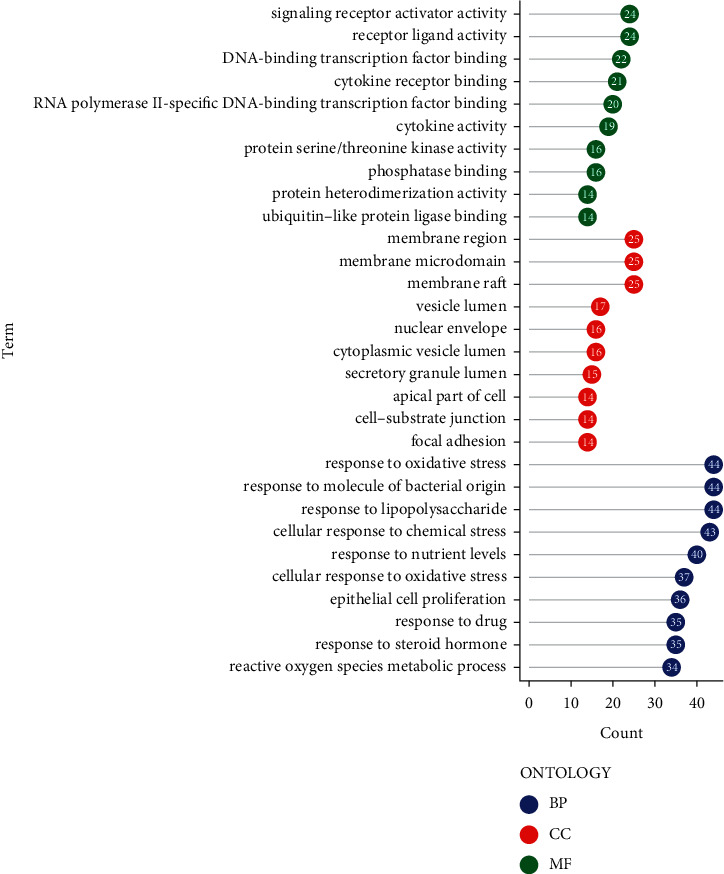
GO enrichment analysis of common targets.

**Figure 4:**
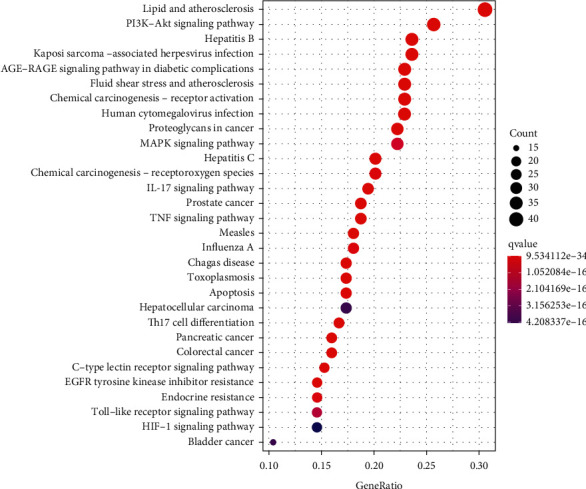
KEGG pathway enrichment analysis of common targets.

GO enrichment analysis revealed 2933 biological functions with remarkable significance, including 2687 for biological processes (BP), 75 for cellular component (CC), and 171 for molecular function (MF). The results of GO enrichment analysis showed that the common targets of SLBZP in treating BA and AC mainly involved response to oxidative stress, response to molecule of bacterial origin, membrane region, membrane microdomain, signaling receptor activator activity, receptor ligand activity, and other biological functions (Figure 3). 182 significant pathways were obtained by KEGG pathway enrichment analysis, mainly involving PI3K-Akt signaling pathway, proteoglycans in cancer, MAPK signaling pathway, IL-17 signaling pathway, TNF signaling pathway, apoptosis, Th17 cell differentiation, and other pathways related to inflammation, cancer, apoptosis, and immunity (Figure 4).

### 3.5. Construction and Analysis of Active Compound-Key Target Network

**Figure 5:**
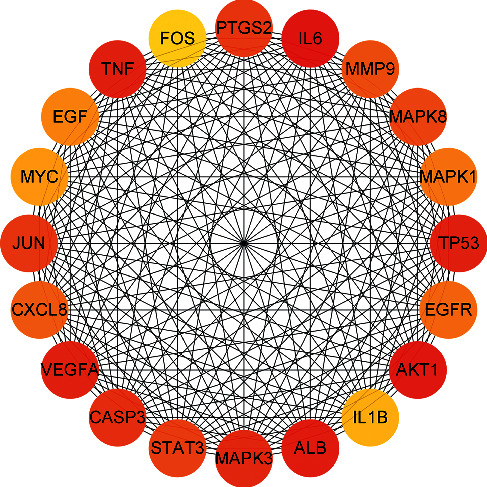
PPI diagram of key targets.

**Figure 6:**
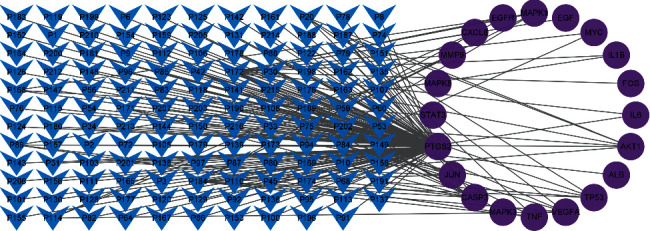
Diagram of active compound-key target network.

The 149 common targets obtained above were screened by “cytoHubba”, a plug-in of Cytoscape software, and then the 20 key targets with the highest degree value were obtained (Figure 5). These 20 key targets and their corresponding active compounds were imported into Cytoscape software for network construction and visualization (Figure 6). There were 131 active compounds related to key targets (Table 3). In the active compound-key target network, the degree of the network topology analysis carried out by “Network Analysis” reflects the connectivity of nodes that respectively represent active compounds and key targets. A higher degree value indicates more associations between nodes, which explains the significances of active compounds and key targets. The results of network topology analysis showed that the 5 active compounds most connected to the key targets were quercetin, luteolin, beta-carotene, kaempferol, and naringenin, and the top 6 key targets of connectivity were prostaglandin G/H synthase 2 (PTGS2), caspase-3 (CASP3), RAC-alpha serine/threonine-protein kinase (AKT1), transcription factor AP-1 (JUN) [, cellular tumor antigen p53 (TP53), and vascular endothelial growth factor A (VEGFA), which indicated that the above compounds and targets were critical and had important implications in SLBZP for treating BA and AC.

### 3.6. Molecular Docking Results

**Figure 7:**
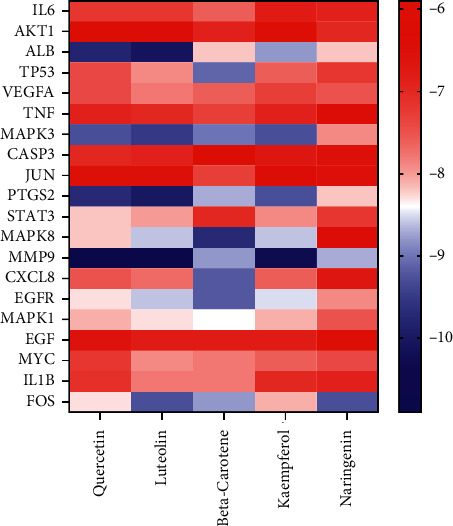
Heat map of docking results between key targets and important active compounds.

**Figure 8:**
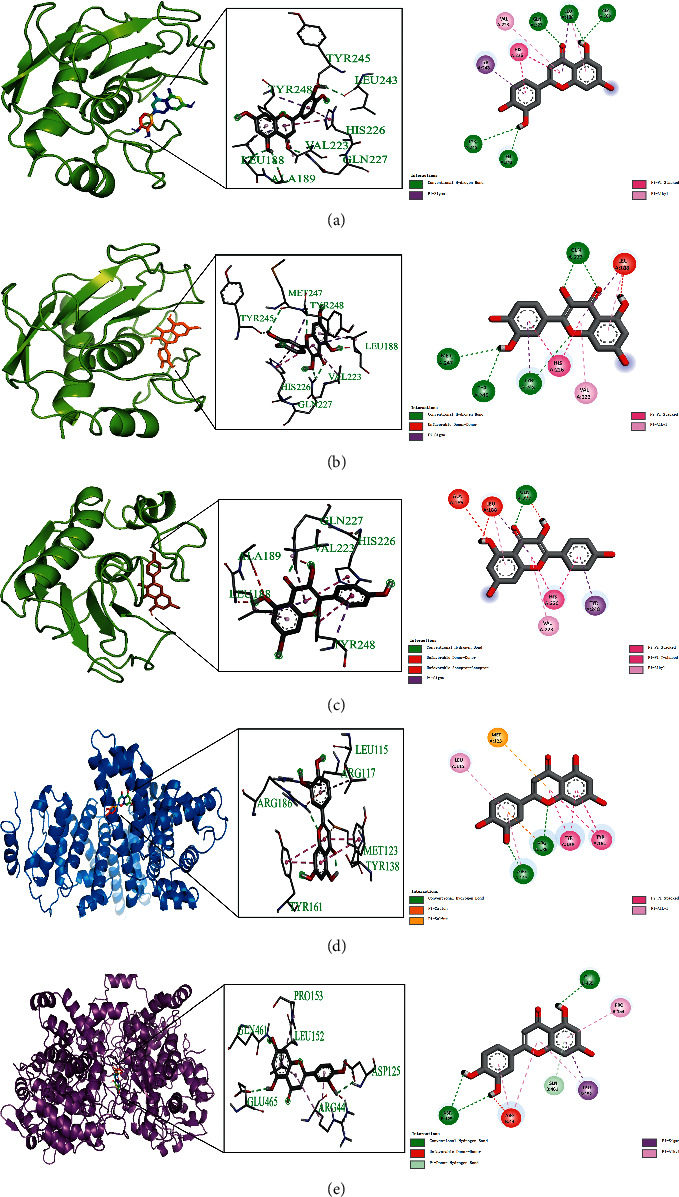
3D and 2D diagrams of molecular docking. (a) MMP9 (6ESM) and luteolin. (b) MMP9 (6ESM) and quercetin. (c) MMP9 (6ESM) and kaempferol. (d) ALB (6YG9) and luteolin. (e) PTGS2 (5F19) and luteolin.

Based on the above analysis results, the 5 important active compounds (quercetin, luteolin, beta-carotene, kaempferol, and naringenin) and the key targets were docked by AutoDockVina software. The docking results are shown in Table 4 and Figure 7. The smaller the binding free energy value, the lower the energy required for binding, which is more conducive to the binding of ligand and protein. Among them, the docking results of MMP9 with luteolin, quercetin, and kaempferol, ALB with luteolin, and PTGS2 with luteolin were the best, as shown in Figure 8. For example, luteolin formed conventional hydrogen bonds with MMP9 protein structure 6ESM amino acid residues A chain TYR245, LEU243, GLN227, LEU188, ALA189, formed *π*-σ interactions with amino acid residues A chain TYR248 and LEU188, formed *π*-*π* stacked interactions with amino acid residue A chain HIS226, and formed *π*-alkyl interactions with amino acid residues A chain VAL223 and LEU188. These forces reduced the binding energy and increased the affinity, which played an auxiliary role in the binding of compound ligand molecules to the residues of target protein structures.

### 3.7. Construction and Analysis of Key Target-miRNA Network

**Figure 9:**
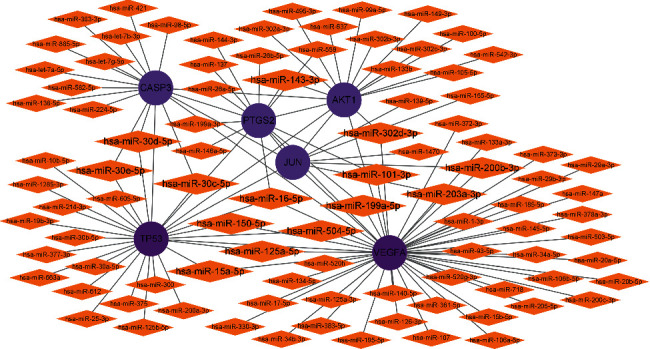
Diagram of key target-miRNA network.

94 miRNAs were predicted from 6 key targets by the miRTarBase database. Cytoscape software was used to construct a key target-miRNA network (Figure 9), among which hsa-miR-16-5p, hsa-miR-101-3p, hsa-miR-143-3p, hsa-miR-199a-5p, hsa-miR-30d-5p, hsa-miR-30c-5p, hsa-miR-30e-5p, hsa-miR-302d-3p, hsa-miR-203a-3p, hsa-miR-200b-3p, hsa-miR-125a-5p, hsa-miR-15a-5p, hsa-miR-504-5p, and hsa-miR-150-5p all targeted multiple key targets.

## 4. Discussion

The theory of traditional Chinese medicine believes that the spleen is the foundation of acquired life and that the spleen is not harmonious and causes all kinds of diseases. Therefore, it has always paid attention to regulating the spleen to protect the five internal organs. The pathogenesis of spleen deficiency is involved in the occurrence and development of BA and AC. SLBZP, one of the classic Earth-cultivating and gold-generating prescriptions, can not only treat BA and AC with simultaneous treatment of different diseases but also protect the spleen to prevent and promote recovery. This study aimed to explore the action mechanism of SLBZP in treating BA and AC with simultaneous treatment of different diseases by using network pharmacology and molecular docking, so as to provide references for more in-depth experimental research and wider clinical applications.

GO annotation results showed that the biological functions involved in common targets were mainly response to oxidative stress, response to molecule of bacterial origin, membrane region, membrane microdomain, signaling receptor activator activity, receptor ligand activity, and so on. In addition, the main enrichment pathways of common targets were PI3K-Akt signaling pathway, proteoglycans in cancer, MAPK signaling pathway, IL-17 signaling pathway, TNF signaling pathway, apoptosis, Th17 cell differentiation, and other pathways related to inflammation, cancer, apoptosis, and immunity. Studies pointed out that, during the onset of asthma, both PI3K-Akt signaling pathway and MAPK signaling pathway were active [35, 36]. Many targets of the PI3K pathway play critical roles in the expression and activation of inflammatory mediators, inflammatory cell recruitment, immune cell function, airway remodeling, and corticosteroid insensitivity in chronic inflammatory airway disease [37]. There were evidences that selective PI3K inhibitors could reduce inflammation and some characteristics of diseases such as abnormal proliferation of airway smooth muscle cells (ASMC) in experimental animal models, which strongly supported that PI3K/Akt inhibitors might be a useful new therapy for asthma [37, 38]. In recent years, many studies confirmed that inhibiting PI3K−Akt signaling pathway and MAPK signaling pathway could effectively inhibit allergic airway inflammation, ASMC proliferation and migration, and phenotypic switching, so as to alleviate airway remodeling and airway hyperresponsiveness (AHR) in asthma [39–42]. Additionally, upregulation of dual-specificity phosphatase-1 (DUSP1), a negative regulator in the MAPK signaling pathway, to healthy levels and downregulation of inflammatory MAPKs at the gene and protein levels could reduce the prevalence of childhood asthma [43]. Proteoglycans enhanced deposition in the airway walls of asthmatics playing a role in airway remodeling, and the difference of deposition in the airway smooth muscle layer of moderate and severe asthmatic patients might affect the functional behavior of airway smooth muscle [44, 45]. IL-17A in the IL-17 signaling pathway was positively correlated with neutrophil accumulation, mucus secretion, macrophage mobilization, and smooth muscle reactivity in various experimental airway models, as well as with disease severity, suggesting that specifically targeting IL-17A had the potential of clinical utility in patients with moderate and severe asthma and high reversibility [46]. Moreover, the reduction of skin inflammation and airway inflammation in the IL-17-induced mouse asthma model was related to the reduction of IL-17-mediated mRNA stability [47]. In TLR ligand-mediated allergic airway inflammation, TLR ligand induced TNF to send signals through airway epithelial cells to promote the development of Th2 in lymph nodes, and TNF was also indispensable in the allergen stimulation stage of neutrophilic and eosinophilic airway inflammation and AHR [48]. Activated TNF-TNFR2 signal transduction could inhibit the differentiation of Th2 and Th17 cells to alleviate allergic airway inflammation [49]. Bronchial cell apoptosis could be observed in some airway biopsies from asthmatic patients, especially those with serious diseases, possibly resulting in airway damage, and dysregulation of leukocyte, eosinophil, and neutrophil apoptosis could lead to asthmatic airway inflammation and was related to the pathogenesis of asthma [50]. Th17 cells, a potent and unique subset that modulated primary bronchial epithelial cell function, were related to the development and pathophysiology of asthma [51, 52]. A study found that asthma-associated IL4R variants promoted the transformation of regulatory T cells into TH17-like cells, thereby exacerbating airway inflammation [53]. It should be noted that because there have been relatively few studies related to allergic colitis all the time, there is almost no relevant research report on the relationship between the above signaling pathways and allergic colitis. However, it is worth mentioning that if further research is carried out on this basis in the future, it will be very innovative and instructive for clarifying the pathogenesis of allergic colitis and developing new drugs that can effectively target the disease. The above showed that SLBZP treated BA and AC with simultaneous treatment of different diseases by multiple functions and pathways, suggesting that further research in the future could be based on these biological functions and pathways, which had guiding significances.

The active compound-key target network of this study showed that the five active compounds of quercetin, luteolin, beta-carotene, kaempferol, and naringenin, and the 6 key targets of PTGS2, CASP3, AKT1, JUN, TP53, and VEGFA were particularly important. Moreover, the results of molecular docking also verified that these five active compounds had good binding characteristics with their corresponding important key targets, indicating that they played vital roles in SLBZP for treating BA and AC with simultaneous treatment of different diseases and had critical potential research values.

Studies suggested that quercetin was known for its antioxidant activity in free radicals scavenging and antiallergic properties [54]. It is characterized by stimulating the immune system and antiviral activity, inhibiting histamine release, reducing proinflammatory cytokines, and producing leukotrienes [55]. It was reported to improve Th1/Th2 balance, inhibit the formation of antigen-specific IgE antibodies, and also be effective in inhibiting enzymes such as lipoxygenase, eosinophils, and peroxidase and inflammatory mediators [56]. All the mentioned mechanisms contribute to the anti-inflammatory and immunomodulatory properties of quercetin, which can be effectively used to treat advanced bronchial asthma, allergic rhinitis, and restrictive allergic reactions caused by peanuts [55]. Luteolin, having anti-inflammatory, antiallergic, and immune-enhancing functions, can reduce airway inflammation and allergies in asthma and has antiallergic effects in mouse models of allergic asthma and rhinitis, which has shown therapeutic effects in treating inflammatory diseases, allergies, bronchial asthma, and systemic damage caused by free radicals [57–59]. It was reported to block the activation of MAPK and NF-*κ*B signaling pathways to protect ARPE-19 cells from the proliferation of IL-6, IL-8, sICAM-1, and MCP-1 stimulated by IL-1*β*, thereby alleviating the inflammatory response [60]. Kaempferol, having antioxidant, anti-inflammatory, anticancer, and antidiabetic effects, could effectively improve allergic and inflammatory airway diseases by interfering with NF-*κ*B signal transduction, which may help alleviate the inflammatory response associated with Cox2 expression [61–63]. Naringenin, having immunomodulatory, anticancer, antimutation, anti-inflammatory, antioxidant, antiproliferative, antiarthritis, and anticarcinogenic effects, can be used for treating osteoporosis, cancer, cardiovascular disease, and rheumatoid arthritis, which exhibits lipid-lowering and insulin-like properties, can inhibit allergen-induced airway inflammation and airway responsiveness, and inhibit NF-*κ*B activity in a mouse model of asthma [64–66]. The above results indicate that SLBZP can fully exert its therapeutic effect by the synergy of multiple compounds, multiple targets, and multiple pathways and provide more new clues for the development of traditional Chinese medicine monomers to treat BA and AC. In addition, the effects of beta-carotene in treating BA and AC are currently seldom studied and reported, which can be used as a direction for in-depth research in the future.

PTGS2, as the most critical target in the network, is one of the key factors of cell response to inflammation and has long been considered to play a key role in the pathogenesis of respiratory inflammation, including asthma [67, 68]. In addition to its anti-inflammatory effect, it can also exert anti-inflammatory/bronchial protection functions in the airway and can be expressed quickly and powerfully in response to various proinflammatory cytokines and mediators [68]. Caspase-3 is necessary for the development of various tissues, playing an important role in neurogenesis, synaptic activity, neuron growth cone guidance, and glial development. It was reported to mediate many nonapoptotic functions in cells and cell death in the process of apoptosis, participate in T and B cell homeostasis in a way that did not depend on apoptosis, and protect compressed organs from cell death [69]. AKT1 ablation promoted the polarization of macrophage M1, which could affect the severity of inflammatory diseases, such as inflammatory bowel disease, and was related to the regulation of innate immunity and inflammation [70]. JUN, the activation of which is caused by the imbalance of pulmonary oxidation and antioxidation in asthma, is an important therapeutic target for allergic airway inflammation and a key transcription factor for the anti-inflammatory activity of dexamethasone and may be an important molecular mechanism of steroids in asthma and other chronic inflammatory lung diseases [71, 72]. As an important mediator of oncogenic *β*-catenin signaling in the intestine, JUN is not only involved in inflammatory response and tumorigenesis but is also related to the inflammatory response in mice with LPS-induced macrophages and DSS-induced colitis [73, 74]. TP53, as a tumor suppressor protein, can produce anti-inflammatory reactions in the lungs and has a potential therapeutic effect in pneumonia, whose dysfunction is associated with acute lung injury, acute respiratory distress syndrome, chronic obstructive pulmonary disease, pulmonary fibrosis, bronchial asthma, pulmonary hypertension, pneumonia and tuberculosis, and so on [75]. It often mutates in human cancers. After the mutations, it prolongs the activation of NF-*κ*B and promotes chronic inflammation and inflammation-related colorectal cancer, which is also related to the occurrence and development of inflammatory bowel disease [76–78]. VEGFA plays a fundamental role in the physiological and pathophysiological forms of angiogenesis. During airway growth, the balance regulation of angiogenic growth factor and vascular inhibitory protein enables the lung to obtain rich blood supply [79]. However, during chronic inflammation, VEGF stimulates angiogenesis and edema and induces Th2 and eosinophilic inflammation, mucous metaplasia, subepithelial fibrosis, myocyte proliferation, and dendritic cell activation, which is a sign of asthma exacerbation and can be used as a target for treating lung diseases and inflammatory bowel diseases [79–82]. The above studies indicate that these six key targets deserve attention in the study of the molecular mechanism of SLBZP for treating BA and AC with simultaneous treatment of different diseases and can be used as potential research objects.

The key target-miRNA network shows that hsa-miR-16-5p, hsa-miR-101-3p, hsa-miR-143-3p, hsa-miR-199a-5p, hsa-miR-30d-5p, hsa-miR-30c-5p, hsa-miR-30e-5p, hsa-miR-302d-3p, hsa-miR-203a-3p, hsa-miR-200b-3p, hsa-miR-125a-5p, hsa-miR-15a-5p, hsa-miR-504-5p, and hsa-miR-150-5p all target and regulate multiple key targets, which may have important upstream regulatory effects and are of great significance for the occurrence, development, and treatment of BA and AC. Studies suggested that baseline airway secretion signatures of hsa-miR-302d-3p and hsa-miR-612 were detected during rhinovirus (RV) infection that was the most common cause of asthma exacerbation and the most important early risk factor for asthma development after childhood in children, which was helpful to develop novel strategies for treating and monitoring respiratory conditions in all age groups [83]. The low tissue level of hsa-miR-200b-3p is related to the cytopathic inflammation caused by human cytomegalovirus infection [84]. Hsa-miR-15a-5p may play an important role in reducing retinal leukopenia by inhibiting inflammatory cell signals, which can be used as a potential target for the inhibition of inflammatory mediators in diabetic retinopathy [85]. In addition to these miRNAs that could target and regulate multiple key targets, hsa-miR-146a-5p, as one of the predicted miRNAs, was upregulated in asthmatic patients to inhibit the expression level of PDE7A, which might be involved in mediating the pathogenesis of asthma [86]. Upregulation of Hsa_circ_0005519 could inhibit the expression of has-let-7a-5p in CD4 T cells of asthmatic patients and promote the production of IL-13 and IL-6, thereby exacerbating asthma [87]. Combining hsa-miR-155-5p and has-miR-532-5p could predict changes in asthma budesonide (ICS) treatment response over time [88]. In allergic settings, the expressions of hsa-miR-139-5p and hsa-miR-542-3p significantly decreased, resulting in increasing expression of pro-inflammatory and antiviral response genes, which might be important during asthma exacerbations [89]. Hsa-miR-19b-3p decreased in the plasma of BA patients, and the ROC curve showed that it could be used as a biomarker for the diagnosis of BA [90]. Hsa-miR-20a-5p, one of the dysregulated miRNAs in asthmatic patients, targeted and inhibited the expression of HDAC4, suppressed the expressions of TNF-*α*, IL-1*β*, and IFN-*γ*, and promoted the production of IL-10, thereby reducing allergic inflammation [91]. Downregulation of hsa-miR-145-5p that increased airway smooth muscle cell proliferation was a risk factor for an early decline (ED) pattern of lung function growth in asthmatic children with chronic obstructive pulmonary disease (COPD) [92]. Once again, it is particularly noted that, at present, there are basically no relevant research reports on these predicted miRNAs related to AC, and there are also very few research reports on these predicted miRNAs related to BA, which means that this study not only provides new insights for in-depth understanding of the pathogenesis of BA and AC and formulating corresponding new treatment strategies but also provides a practical basis for future validation studies. In general, there are few reports on the above-mentioned miRNAs that have great research potentials.

## 5. Conclusions

In conclusion, network pharmacology and molecular docking technology demonstrated that SLBZP in treating BA and AC with simultaneous treatment of different diseases was a complex process involving multiple compounds, multiple targets, and multiple pathways. It may involve important active compounds and key targets represented by quercetin, luteolin, beta-carotene, kaempferol, naringenin, PTGS2, CASP3, AKT1, JUN, TP53, and VEGFA, may be related to inflammation, cancer, apoptosis, and immune-related pathways, and may involve the targeted regulation of multiple upstream miRNAs. These can provide references for future clinical and experimental studies.

## Figures and Tables

**Table 1:** Characteristics of eligible active compounds in SLBZP with OB and DL parameters.

Code	Molecule ID	Molecule name	OB (%)	DL	Herbs
P1	MOL004924	(-)-Medicocarpin	40.99	0.95	Gancao
P2	MOL004988	Kanzonol F	32.47	0.89	Gancao
P3	MOL005018	Xambioona	54.85	0.87	Gancao
P4	MOL005458	Dioscoreside C_qt	36.38	0.87	Shanyao
P5	MOL007536	Stigmasta-5, 22-dien-3-beta-yl acetate	46.44	0.86	Sharen
P6	MOL001474	Sanguinarine	37.81	0.86	Sharen
P7	MOL001973	Sitosteryl acetate	40.39	0.85	Sharen
P8	MOL004948	Isoglycyrol	44.7	0.84	Gancao
P9	MOL008752	Dihydroverticillatine	42.69	0.84	Jiegeng
P10	MOL000787	Fumarine	59.26	0.83	Renshen
P11	MOL005357	Gomisin B	31.99	0.83	Renshen
P12	MOL000300	Dehydroeburicoic acid	44.17	0.83	Fuling
P13	MOL000285	(2R)-2-[(5R, 10S, 13R, 14R, 16R, 17R)-16-hydroxy-3-keto-4, 4, 10, 13, 14-pentamethyl-1, 2, 5, 6, 12, 15, 16, 17-octahydrocyclopenta[a]phenanthren-17-yl]-5-isopropyl-hex-5-enoic acid	38.26	0.82	Fuling
P14	MOL000280	(2R)-2-[(3S, 5R, 10S, 13R, 14R, 16R, 17R)-3, 16-dihydroxy-4, 4, 10, 13, 14-pentamethyl-2, 3, 5, 6, 12, 15, 16, 17-octahydro-1h-cyclopenta[a]phenanthren-17-yl]-5-isopropyl-hex-5-enoic acid	31.07	0.82	Fuling
P15	MOL005317	Deoxyharringtonine	39.27	0.81	Renshen
P16	MOL000283	Ergosterol peroxide	40.36	0.81	Fuling
P17	MOL000287	3beta-hydroxy-24-methylene-8-lanostene-21-oic acid	38.7	0.81	Fuling
P18	MOL000276	7, 9(11)-Dehydropachymic acid	35.11	0.81	Fuling
P19	MOL000289	Pachymic acid	33.63	0.81	Fuling
P20	MOL000546	Diosgenin	80.88	0.81	Shanyao
P21	MOL000275	Trametenolic acid	38.71	0.80	Fuling
P22	MOL005376	Panaxadiol	33.09	0.79	Renshen
P23	MOL005401	Ginsenoside Rg5_qt	39.56	0.79	Renshen
P24	MOL004917	Glycyroside	37.25	0.79	Gancao
P25	MOL007535	(5S, 8S, 9S, 10R, 13R, 14S, 17R)-17-[(1R, 4R)-4-ethyl-1, 5-dimethylhexyl]-10, 13-dimethyl-2, 4, 5, 7, 8, 9, 11, 12, 14, 15, 16, 17-dodecahydro-1h-cyclopenta[a]phenanthrene-3, 6-dione	33.12	0.79	Sharen
P26	MOL005348	Ginsenoside-Rh4_qt	31.11	0.78	Renshen
P27	MOL000033	(3S, 8S, 9S, 10R, 13R, 14S, 17R)-10, 13-dimethyl-17-[(2R, 5S)-5-propan-2-yloctan-2-yl]-2, 3, 4, 7, 8, 9, 11, 12, 14, 15, 16, 17-dodecahydro-1h-cyclopenta[a]phenanthren-3-ol	36.23	0.78	Baizhu
P28	MOL009136	Peraksine	82.58	0.78	Fuling
P29	MOL000211	Mairin	55.38	0.78	Gancao
P30	MOL005001	Gancaonin H	50.10	0.78	Gancao
P31	MOL001323	Sitosterol alpha1	43.28	0.78	Yiyiren
P32	MOL000279	Cerevisterol	37.96	0.77	Fuling
P33	MOL005465	AIDS180907	45.33	0.77	Shanyao
P34	MOL000449	Stigmasterol	43.83	0.76	Renshen, yiyiren, sharen, baibiandou, shanyao
P35	MOL000028	*α*-Amyrin	39.51	0.76	Baizhu
P36	MOL000290	Poricoic acid A	30.61	0.76	Fuling
P37	MOL001755	24-Ethylcholest-4-en-3-one	36.08	0.76	Renshen
P38	MOL004355	Spinasterol	42.98	0.76	Jiegeng
P39	MOL004718	*α*-Spinasterol	42.98	0.76	Jiegeng
P40	MOL005440	Isofucosterol	43.78	0.76	Shanyao
P41	MOL010625	24-Methylenecholesterol	43.54	0.76	Shanyao
P42	MOL000358	Beta-sitosterol	36.91	0.75	Renshen, sharen
P43	MOL005399	Alexandrin_qt	36.91	0.75	Renshen
P44	MOL001525	Daucosterol	36.91	0.75	Renshen
P45	MOL000296	Hederagenin	36.91	0.75	Fuling
P46	MOL000292	Poricoic acid C	38.15	0.75	Fuling
P47	MOL000291	Poricoic acid B	30.52	0.75	Fuling
P48	MOL006376	7-Dehydrosigmasterol	37.42	0.75	Fuling
P49	MOL000359	Sitosterol	36.91	0.75	Gancao, yiyiren
P50	MOL001771	Poriferast-5-en-3beta-ol	36.91	0.75	Sharen
P51	MOL013119	Enhydrin	40.56	0.74	Renshen
P52	MOL000139	Smitilbin	37.60	0.74	Renshen
P53	MOL009387	Didehydrotuberostemonine	51.91	0.74	Baizhu
P54	MOL004903	Liquiritin	65.69	0.74	Gancao
P55	MOL009154	Tuberostemoenone	53.90	0.73	Baizhu
P56	MOL004891	Shinpterocarpin	80.30	0.73	Gancao
P57	MOL009431	Stemonine	81.75	0.72	Baizhu
P58	MOL000282	Ergosta-7, 22e-dien-3beta-ol	43.51	0.72	Fuling
P59	MOL009149	Cheilanthifoline	46.51	0.72	Fuling
P60	MOL004805	(2S)-2-[4-hydroxy-3-(3-methylbut-2-enyl)phenyl]-8, 8-dimethyl-2, 3-dihydropyrano[2, 3-f]chromen-4-one	31.79	0.72	Gancao
P61	MOL005435	24-Methylcholest-5-enyl-3belta-O-glucopyranoside_qt	37.58	0.72	Shanyao
P62	MOL012254	Campesterol	37.58	0.71	Renshen
P63	MOL005438	Campesterol	37.58	0.71	Renshen, shanyao
P64	MOL000493	Campesterol	37.58	0.71	Renshen
P65	MOL005013	18 *α*-Hydroxyglycyrrhetic acid	41.16	0.71	Gancao
P66	MOL006070	Robinin	39.84	0.71	Jiegeng
P67	MOL011042	18Alpha-hydroglycyrrhetic acid	38.93	0.71	Baibiandou
P68	MOL004567	Isoengelitin	34.65	0.70	Renshen
P69	MOL007180	Vitamin-e	32.29	0.70	Sharen
P70	MOL000953	CLR	37.87	0.68	Yiyiren, shanyao
P71	MOL000554	Gallic acid-3-O-(6′-O-galloyl)-glucoside	30.25	0.67	Fuling, sharen
P72	MOL002311	Glycyrol	90.78	0.67	Gancao
P73	MOL011455	20-Hexadecanoylingenol	32.70	0.65	Renshen, fuling
P74	MOL004904	Licopyranocoumarin	80.36	0.65	Gancao
P75	MOL004959	1-Methoxyphaseollidin	69.98	0.64	Gancao
P76	MOL004071	Hyndarin	73.94	0.64	Gancao
P77	MOL005360	Malkangunin	57.71	0.63	Renshen, baizhu
P78	MOL004824	(2S)-6-(2, 4-dihydroxyphenyl)-2-(2-hydroxypropan-2-yl)-4-methoxy-2, 3-dihydrofuro[3, 2-g]chromen-7-one	60.25	0.63	Gancao
P79	MOL005008	Glycyrrhiza flavonol A	41.28	0.60	Gancao
P80	MOL005007	Glyasperins M	72.67	0.59	Gancao
P81	MOL004492	Chrysanthemaxanthin	38.72	0.58	Renshen, fuling
P82	MOL005017	Phaseol	78.77	0.58	Gancao
P83	MOL005003	Licoagrocarpin	58.81	0.58	Gancao
P84	MOL002773	Beta-carotene	37.18	0.58	Baibiandou
P85	MOL004974	3′-methoxyglabridin	46.16	0.57	Gancao
P86	MOL004966	3′-hydroxy-4′-O-Methylglabridin	43.71	0.57	Gancao
P87	MOL004806	Euchrenone	30.29	0.57	Gancao
P88	MOL005384	Suchilactone	57.52	0.56	Renshen, baizhu
P89	MOL005344	Ginsenoside rh2	36.32	0.56	Renshen
P90	MOL006982	Codeine	45.48	0.56	Sharen
P91	MOL004827	Semilicoisoflavone B	48.78	0.55	Gancao
P92	MOL004884	Licoisoflavone B	38.93	0.55	Gancao
P93	MOL004905	3, 22-Dihydroxy-11-oxo-delta(12)-oleanene-27-alpha-methoxycarbonyl-29-oic acid	34.32	0.55	Gancao
P94	MOL003648	Inermine	65.83	0.54	Renshen
P95	MOL004810	Glyasperin F	75.84	0.54	Gancao
P96	MOL001484	Inermine	75.18	0.54	Gancao
P97	MOL004885	Licoisoflavanone	52.47	0.54	Gancao
P98	MOL005461	Doradexanthin	38.16	0.54	Shanyao
P99	MOL004914	1, 3-Dihydroxy-8, 9-dimethoxy-6-benzofurano [3, 2-c]chromenone	62.90	0.53	Gancao
P100	MOL004820	Kanzonols W	50.48	0.52	Gancao
P101	MOL004978	2-[(3R)-8, 8-Dimethyl-3, 4-dihydro-2h-pyrano [6, 5-f]chromen-3-yl]-5-methoxyphenol	36.21	0.52	Gancao
P102	MOL003851	Isoramanone	39.97	0.51	Gancao
P103	MOL004912	Glabrone	52.51	0.50	Gancao
P104	MOL005314	Celabenzine	101.88	0.49	Renshen
P105	MOL005012	Licoagroisoflavone	57.28	0.49	Gancao
P106	MOL004855	Licoricone	63.58	0.47	Gancao
P107	MOL004908	Glabridin	53.25	0.47	Gancao
P108	MOL004879	Glycyrin	52.61	0.47	Gancao
P109	MOL009436	Stemotinine	38.69	0.46	Baizhu
P110	MOL004857	Gancaonin B	48.79	0.45	Gancao
P111	MOL004833	Phaseolinisoflavan	32.01	0.45	Gancao
P112	MOL004808	Glyasperin B	65.22	0.44	Gancao
P113	MOL004911	Glabrene	46.27	0.44	Gancao
P114	MOL001002	Ellagic acid	43.06	0.43	Fuling, sharen
P115	MOL004849	3-(2, 4-Dihydroxyphenyl)-8-(1, 1-dimethylprop-2-enyl)-7-hydroxy-5-methoxy-coumarin	59.62	0.43	Gancao
P116	MOL004913	1, 3-Dihydroxy-9-methoxy-6-benzofurano [3, 2-c]chromenone	48.14	0.43	Gancao
P117	MOL008118	Coixenolide	32.40	0.43	Yiyiren
P118	MOL004949	Isolicoflavonol	45.17	0.42	Gancao
P119	MOL004883	Licoisoflavone	41.61	0.42	Gancao
P120	MOL004814	Isotrifoliol	31.94	0.42	Gancao
P121	MOL002372	(6Z, 10E, 14E, 18E)-2, 6, 10, 15, 19, 23-Hexamethyltetracosa-2, 6, 10, 14, 18, 22-hexaene	33.55	0.42	Yiyiren
P122	MOL004863	3-(3, 4-Dihydroxyphenyl)-5, 7-dihydroxy-8-(3-methylbut-2-enyl)chromone	66.37	0.41	Gancao
P123	MOL004866	2-(3, 4-Dihydroxyphenyl)-5, 7-dihydroxy-6-(3-methylbut-2-enyl)chromone	44.15	0.41	Gancao
P124	MOL004989	6-Prenylated eriodictyol	39.22	0.41	Gancao
P125	MOL004935	Sigmoidin-B	34.88	0.41	Gancao
P126	MOL004864	5, 7-Dihydroxy-3-(4-methoxyphenyl)-8-(3-methylbut-2-enyl)chromone	30.49	0.41	Gancao
P127	MOL005890	Pachypodol	75.06	0.40	Fuling
P128	MOL004993	8-Prenylated eriodictyol	53.79	0.40	Gancao
P129	MOL004856	Gancaonin A	51.08	0.40	Gancao
P130	MOL004811	Glyasperin C	45.56	0.40	Gancao
P131	MOL007213	Nuciferine	34.43	0.40	Lianzi
P132	MOL012537	Spinoside A	41.75	0.40	Jiegeng
P133	MOL008406	Spinoside A	39.97	0.40	Jiegeng
P134	MOL002879	Diop	43.59	0.39	Renshen
P135	MOL005000	Gancaonin G	60.44	0.39	Gancao
P136	MOL005430	Hancinone C	59.05	0.39	Shanyao
P137	MOL004838	8-(6-Hydroxy-2-benzofuranyl)-2, 2-dimethyl-5-chromenol	58.44	0.38	Gancao
P138	MOL006980	Papaverine	64.04	0.38	Sharen
P139	MOL000322	Kadsurenone	54.72	0.38	Shanyao
P140	MOL000310	Denudatin B	61.47	0.38	Shanyao
P141	MOL005020	Dehydroglyasperins C	53.82	0.37	Gancao
P142	MOL003656	Lupiwighteone	51.64	0.37	Gancao
P143	MOL004915	Eurycarpin A	43.28	0.37	Gancao
P144	MOL009172	Pronuciferin	32.75	0.37	Lianzi
P145	MOL005429	Hancinol	64.01	0.37	Shanyao
P146	MOL004882	Licocoumarone	33.21	0.36	Gancao
P147	MOL003673	Wighteone	42.80	0.36	Gancao
P148	MOL004907	Glyzaglabrin	61.07	0.35	Gancao
P149	MOL004828	Glepidotin A	44.72	0.35	Gancao
P150	MOL004815	(E)-1-(2, 4-dihydroxyphenyl)-3-(2, 2-dimethylchromen-6-yl)prop-2-en-1-one	39.62	0.35	Gancao
P151	MOL005321	Frutinone A	65.90	0.34	Renshen
P152	MOL004829	Glepidotin B	64.46	0.34	Gancao
P153	MOL002565	Medicarpin	49.22	0.34	Gancao
P154	MOL011072	Quinicine	75.44	0.33	Fuling, baibiandou
P155	MOL004961	Quercetin der.	46.45	0.33	Gancao
P156	MOL004980	Inflacoumarin A	39.71	0.33	Gancao
P157	MOL004848	Licochalcone G	49.25	0.32	Gancao
P158	MOL004945	(2S)-7-hydroxy-2-(4-hydroxyphenyl)-8-(3-methylbut-2-enyl)chroman-4-one	36.57	0.32	Gancao
P159	MOL005356	Girinimbin	61.22	0.31	Renshen
P160	MOL000021	14-Acetyl-12-senecioyl-2E, 8E, 10E-atractylentriol	60.31	0.31	Baizhu
P161	MOL004910	Glabranin	52.90	0.31	Gancao
P162	MOL000354	Isorhamnetin	49.60	0.31	Gancao, baibiandou
P163	MOL004898	(E)-3-[3, 4-dihydroxy-5-(3-methylbut-2-enyl)phenyl]-1-(2, 4-dihydroxyphenyl)prop-2-en-1-one	46.27	0.31	Gancao
P164	MOL000022	14-Acetyl-12-senecioyl-2E, 8Z, 10E-atractylentriol	63.37	0.30	Baizhu
P165	MOL005016	Odoratin	49.95	0.30	Gancao
P166	MOL002882	[(2R)-2, 3-dihydroxypropyl] (Z)-octadec-9-enoate	34.13	0.30	Yiyiren
P167	MOL000239	Jaranol	50.83	0.29	Gancao
P168	MOL000497	Licochalcone a	40.79	0.29	Gancao
P169	MOL007206	Armepavine	69.31	0.29	Lianzi
P170	MOL008121	2-Monoolein	34.23	0.29	Yiyiren
P171	MOL009135	Ellipticine	30.82	0.28	Fuling, sharen
P172	MOL000098	Quercetin	46.43	0.28	Gancao, sharen, baibiandou
P173	MOL004576	Taxifolin	57.84	0.27	Renshen
P174	MOL004990	7, 2′, 4′-Trihydroxy－5-methoxy-3-arylcoumarin	83.71	0.27	Gancao
P175	MOL004860	Licorice glycoside E	32.89	0.27	Gancao
P176	MOL005575	Gentiacaulein	72.82	0.27	Gancao
P177	MOL001735	Dinatin	30.97	0.27	Gancao
P178	MOL004580	cis-Dihydroquercetin	66.44	0.27	Jiegeng
P179	MOL001736	(-)-Taxifolin	60.51	0.27	Shanyao
P180	MOL005267	Elymoclavine	72.87	0.27	Shanyao
P181	MOL004991	7-Acetoxy-2-methylisoflavone	38.92	0.26	Gancao
P182	MOL011093	Apohyoscine	59.68	0.25	Renshen
P183	MOL003617	Isogosferol	30.07	0.25	Gancao
P184	MOL000006	Luteolin	36.16	0.25	Jiegeng
P185	MOL005996	2-O-methyl-3―O-*β*-D-glucopyranosyl platycogenate A	45.15	0.25	Jiegeng
P186	MOL006026	Dimethyl 2-O-methyl-3-O-a-D-glucopyranosyl platycogenate A	39.21	0.25	Jiegeng
P187	MOL000422	Kaempferol	41.88	0.24	Renshen, gancao, baibiandou
P188	MOL000417	Calycosin	47.75	0.24	Gancao
P189	MOL005573	Genkwanin	37.13	0.24	Gancao
P190	MOL000492	(+)-Catechin	54.83	0.24	Sharen, baibiandou
P191	MOL001689	Acacetin	34.97	0.24	Jiegeng
P192	MOL005463	Methylcimicifugoside_qt	31.69	0.24	Shanyao
P193	MOL007514	Methyl icosa-11, 14-dienoate	39.67	0.23	Sharen
P194	MOL003975	Icosa-11, 14, 17-trienoic acid methyl ester	44.81	0.23	Sharen
P195	MOL005308	Aposiopolamine	66.65	0.22	Renshen
P196	MOL000049	3*β*-acetoxyatractylone	54.07	0.22	Baizhu
P197	MOL000020	12-Senecioyl-2E, 8 E, 10E-atractylentriol	62.40	0.22	Baizhu
P198	MOL000072	8*β*-ethoxy atractylenolide III	35.95	0.21	Baizhu
P199	MOL010586	Formononetin	66.39	0.21	Baizhu
P200	MOL000500	Vestitol	74.66	0.21	Gancao
P201	MOL000392	Formononetin	69.67	0.21	Gancao, baibiandou
P202	MOL004328	Naringenin	59.29	0.21	Gancao, yiyiren, jiegeng
P203	MOL004957	HMO	38.37	0.21	Gancao
P204	MOL002419	Demethylcoclaurine((R)-norcoclaurine)	82.54	0.21	Lianzi
P205	MOL005320	Arachidonate	45.57	0.20	Renshen
P206	MOL005318	Dianthramine	40.45	0.20	Renshen
P207	MOL003896	7-Methoxy-2-methyl isoflavone	42.56	0.20	Gancao
P208	MOL004985	Icos-5-enoic acid	30.70	0.20	Gancao
P209	MOL004996	Gadelaidic acid	30.70	0.20	Gancao
P210	MOL000230	Pinocembrin	57.56	0.20	Gancao
P211	MOL004841	Licochalcone B	76.76	0.19	Gancao
P212	MOL004835	Glypallichalcone	61.60	0.19	Gancao
P213	MOL001494	Mandenol	42.00	0.19	Yiyiren
P214	MOL004058	Khell	33.19	0.19	Shanyao
P215	MOL004941	(2R)-7-hydroxy-2-(4-hydroxyphenyl)chroman-4-one	71.12	0.18	Gancao
P216	MOL001792	DFV	32.76	0.18	Gancao
P217	MOL001559	Piperlonguminine	30.71	0.18	Shanyao

**Table 2:** Characteristics of common targets.

No.	Target	Symbol	UniProt ID	No.	Target	Symbol	UniProt ID
1	72 kDa type IV collagenase	MMP2	P08253	76	Prostaglandin E2 receptor EP3 subtype	PTGER3	P43115
2	Xanthine dehydrogenase/oxidase	XDH	P47989	77	Urokinase-type plasminogen activator	PLAU	P00749
3	Heat shock protein beta-1	HSPB1	P04792	78	Phosphatidylinositol-3,4,5-trisphosphate 3-phosphatase and dual-specificity protein phosphatase PTEN	PTEN	P60484
4	Nitric oxide synthase, inducible	NOS2	P35228	79	Sodium-dependent serotonin transporter	SLC6A4	P31645
5	Hepatocyte growth factor receptor	MET	P08581	80	Interferon regulatory factor 1	IRF1	P10914
6	UDP-glucuronosyltransferase 1–1	UGT1A1	P22309	81	Arachidonate 5-lipoxygenase	ALOX5	P09917
7	Protein kinase C beta type	PRKCB	P05771	82	Gap junction alpha-1 protein	GJA1	P17302
8	Collagen alpha-1(I) chain	COL1A1	P02452	83	Claudin-4	CLDN4	O14493
9	Baculoviral IAP repeat-containing protein 5	BIRC5	O15392	84	Dipeptidyl peptidase IV	DPP4	P27487
10	Apoptosis regulator Bcl-2	BCL2	P10415	85	Serum paraoxonase/arylesterase 1	PON1	P27169
11	Alpha-2A adrenergic receptor	ADRA2A	P08913	86	Caspase-8	CASP8	Q14790
12	Cytochrome P450 1A1	CYP1A1	P04798	87	Peroxisome proliferator activated receptor gamma	PPARG	P37231
13	5-Hydroxytryptamine receptor 3A	HTR3A	P46098	88	C-X-C motif chemokine 11	CXCL11	O14625
14	Mitogen-activated protein kinase 10	MAPK10	P53779	89	Interleukin-8	CXCL8	P10145
15	Prostaglandin *E* synthase	PTGES	O14684	90	E-selectin	SELE	P16581
16	C-reactive protein	CRP	P02741	91	Thrombomodulin	THBD	P07204
17	Glutathione S-transferase P	GSTP1	P09211	92	Glucocorticoid receptor	NR3C1	P04150
18	Aryl hydrocarbon receptor	AHR	P35869	93	Serine/threonine-protein kinase mTOR	MTOR	P42345
19	Nuclear factor erythroid 2-related factor 2	NFE2L2	Q16236	94	Mitogen-activated protein kinase 14	MAPK14	Q16539
20	Tumor necrosis factor	TNF	P01375	95	RAF proto-oncogene serine/threonine-protein kinase	RAF1	P04049
21	Pro-epidermal growth factor	EGF	P01133	96	Cytosolic phospholipase A2	PLA2G4A	P47712
22	Interleukin-1 alpha	IL1A	P01583	97	Myeloperoxidase	MPO	P05164
23	Canalicular multispecific organic anion transporter 1	ABCC2	Q92887	98	Alpha-1B adrenergic receptor	ADRA1B	P35368
24	Caspase-1	CASP1	P29466	99	Inhibitor of nuclear factor kappa-B kinase subunit alpha	CHUK	O15111
25	Osteopontin	SPP1	P10451	100	Signal transducer and activator of transcription 3	STAT3	P40763
26	Thrombin	F2	P00734	101	Antileukoproteinase	SLPI	P03973
27	Prostaglandin G/H synthase 2	PTGS2	P35354	102	Cathepsin D	CTSD	P07339
28	Catenin beta-1	CTNNB1	P35222	103	Sterol O-acyltransferase 1	SOAT1	P35610
29	G1/S-specific cyclin-D1	CCND1	P24385	104	Acetylcholinesterase	ACHE	P22303
30	Estrogen receptor	ESR1	P03372	105	Induced myeloid leukemia cell differentiation protein Mcl-1	MCL1	Q07820
31	Vascular endothelial growth factor A	VEGFA	P15692	106	C-C motif chemokine 2	CCL2	P13500
32	Transforming growth factor beta-1	TGFB1	P01137	107	Interleukin-6	IL6	P05231
33	Myc proto-oncogene protein	MYC	P01106	108	Caspase-3	CASP3	P42574
34	Cyclin-A2	CCNA2	P20248	109	Heat shock protein HSP 90-alpha	HSP90AA1	P07900
35	Glycogen synthase kinase-3 beta	GSK3B	P49841	110	Poly [ADP-ribose] polymerase 1	PARP1	P09874
36	Interstitial collagenase	MMP1	P03956	111	Tumor necrosis factor ligand superfamily member 6	FASLG	P48023
37	Signal transducer and activator of transcription 1-alpha/beta	STAT1	P42224	112	Maltase-glucoamylase, intestinal	MGAM	O43451
38	Peroxisome proliferator activated receptor delta	PPARD	Q03181	113	Vascular endothelial growth factor receptor 2	KDR	P35968
39	3-Hydroxy-3-methylglutaryl-coenzyme a reductase	HMGCR	P04035	114	Fos-related antigen 2	FOSL2	P15408
40	Mineralocorticoid receptor	NR3C2	P08235	115	ATP-binding cassette sub-family *G* member 2	ABCG2	Q9UNQ0
41	Glutathione reductase, mitochondrial	GSR	P00390	116	Peroxisome proliferator-activated receptor alpha	PPARA	Q07869
42	Heme oxygenase 1	HMOX1	P09601	117	Cytochrome P450 1A2	CYP1A2	P05177
43	Stromelysin-1	MMP3	P08254	118	Insulin-like growth factor II	IGF2	P01344
44	Pituitary adenylate cyclase-activating polypeptide	ADCYAP1	P18509	119	Phosphatidylinositol-4,5-bisphosphate 3-kinase catalytic subunit, gamma isoform	PIK3CG	P48736
45	Glutathione S-transferase mu 1	GSTM1	P09488	120	NAD(P)H dehydrogenase [quinone] 1	NQO1	P15559
46	Interleukin-10	IL10	P22301	121	Interleukin-2	IL2	P60568
47	Mitogen-activated protein kinase 1	MAPK1	P28482	122	Receptor tyrosine-protein kinase erbB-3	ERBB3	P21860
48	C-X-C motif chemokine 2	CXCL2	P19875	123	Interferon gamma	IFNG	P01579
49	Epidermal growth factor receptor	EGFR	P00533	124	Proto-oncogene c-Fos	FOS	P01100
50	Inhibitor of nuclear factor kappa-B kinase subunit beta	IKBKB	O14920	125	78 kDa glucose-regulated protein	HSPA5	P11021
51	Superoxide dismutase [Cu-Zn]	SOD1	P00441	126	Intercellular adhesion molecule 1	ICAM1	P05362
52	Receptor tyrosine-protein kinase erbB-2	ERBB2	P04626	127	Caveolin-1	CAV1	Q03135
53	Interleukin-4	IL4	P05112	128	Bcl-2-like protein 1	BCL2L1	Q07817
54	Mitogen-activated protein kinase 8	MAPK8	P45983	129	Mitogen-activated protein kinase 3	MAPK3	P27361
55	Aldose reductase	AKR1B1	P15121	130	Carbonic anhydrase II	CA2	P00918
56	Histamine H1 receptor	HRH1	P35367	131	Transcription factor p65	RELA	Q04206
57	Cell division protein kinase 2	CDK2	P24941	132	Hypoxia-inducible factor 1-alpha	HIF1A	Q16665
58	Progesterone receptor	PGR	P06401	133	Nitric-oxide synthase, endothelial	NOS3	P29474
59	Ornithine decarboxylase	ODC1	P11926	134	Mu-type opioid receptor	OPRM1	P35372
60	C-X-C motif chemokine 10	CXCL10	P02778	135	Plasminogen activator inhibitor 1	SERPINE1	P05121
61	Cellular tumor antigen p53	TP53	P04637	136	Vascular cell adhesion protein 1	VCAM1	P19320
62	Caspase-9	CASP9	P55211	137	RAC-alpha serine/threonine-protein kinase	AKT1	P31749
63	Cyclin-dependent kinase inhibitor 1	CDKN1A	P38936	138	Prostaglandin G/H synthase 1	PTGS1	P23219
64	Catalase	CAT	P04040	139	Tissue factor	F3	P13726
65	NAD-dependent deacetylase sirtuin-1	SIRT1	Q96EB6	140	Nuclear receptor sub-family 1 group I member 2	NR1I2	O75469
66	Multidrug resistance-associated protein 1	ABCC1	P33527	141	Transcription factor AP-1	JUN	P05412
67	Interleukin-1 beta	IL1B	P01584	142	Androgen receptor	AR	P10275
68	NF-kappa-B inhibitor alpha	NFKBIA	P25963	143	Apoptosis regulator BAX	BAX	Q07812
69	Insulin-like growth factor-binding protein 3	IGFBP3	P17936	144	Protein kinase C alpha type	PRKCA	P17252
70	Serum albumin	ALB	P02768	145	CD40 ligand	CD40LG	P29965
71	5-Hydroxytryptamine 2A receptor	HTR2A	P28223	146	Cytochrome P450 3A4	CYP3A4	P08684
72	Stromelysin-2	MMP10	P09238	147	Matrix metalloproteinase-9	MMP9	P14780
73	Estrogen receptor beta	ESR2	Q92731	148	Adiponectin	ADIPOQ	Q15848
74	Cytochrome P450 1B1	CYP1B1	Q16678	149	Retinoic acid receptor RXR-beta	RXRB	P28702
75	Neuronal acetylcholine receptor protein, alpha-7 chain	CHRNA7	P36544				

**Table 3:** Active compounds related to key targets.

Code	Molecule ID	Molecule name	OB (%)	DL	Degree	Herbs
P172	MOL000098	Quercetin	46.43	0.28	16	Gancao, sharen, baibiandou
P184	MOL000006	Luteolin	36.16	0.25	11	Jiegeng
P84	MOL002773	Beta-carotene	37.18	0.58	7	Baibiandou
P187	MOL000422	Kaempferol	41.88	0.24	6	Renshen, gancao, baibiandou
P202	MOL004328	Naringenin	59.29	0.21	5	Gancao, yiyiren, jiegeng
P20	MOL000546	Diosgenin	80.88	0.81	4	Shanyao
P89	MOL005344	Ginsenoside rh2	36.32	0.56	4	Renshen
P171	MOL009135	Ellipticine	30.82	0.28	3	Fuling, sharen
P191	MOL001689	Acacetin	34.97	0.24	3	Jiegeng
P114	MOL001002	Ellagic acid	43.06	0.43	3	Fuling, sharen
P42	MOL000358	Beta-sitosterol	36.91	0.75	3	Renshen, sharen
P168	MOL000497	Licochalcone a	40.79	0.29	3	Gancao
P201	MOL000392	Formononetin	69.67	0.21	2	Gancao, baibiandou
P151	MOL005321	Frutinone A	65.90	0.34	1	Renshen
P94	MOL003648	Inermine	65.83	0.54	1	Renshen
P159	MOL005356	Girinimbin	61.22	0.31	1	Renshen
P10	MOL000787	Fumarine	59.26	0.83	1	Renshen
P88	MOL005384	Suchilactone	57.52	0.56	1	Renshen, baizhu
P205	MOL005320	Arachidonate	45.57	0.20	1	Renshen
P34	MOL000449	Stigmasterol	43.83	0.76	1	Shanyao
P206	MOL005318	Dianthramine	40.45	0.20	1	Renshen
P68	MOL004567	Isoengelitin	34.65	0.70	1	Renshen
P173	MOL004576	Taxifolin	57.84	0.27	1	Renshen
P64	MOL000493	Campesterol	37.58	0.71	1	Renshen
P164	MOL000022	14-Acetyl-12-senecioyl-2E,8Z,10E-atractylentriol	63.37	0.30	1	Baizhu
P196	MOL000049	3*β*-acetoxyatractylone	54.07	0.22	1	Baizhu
P198	MOL000072	8*β*-ethoxy atractylenolide III	35.95	0.21	1	Baizhu
P199	MOL010586	Formononetin	66.39	0.21	1	Baizhu
P53	MOL009387	Didehydrotuberostemonine	51.91	0.74	1	Baizhu
P45	MOL000296	Hederagenin	36.91	0.75	1	Fuling
P59	MOL009149	Cheilanthifoline	46.51	0.72	1	Fuling
P154	MOL011072	Quinicine	75.44	0.33	1	Fuling, baibiandou
P72	MOL002311	Glycyrol	90.78	0.67	1	Gancao
P174	MOL004990	7,2′,4′-trihydroxy-5-methoxy-3-arylcoumarin	83.71	0.27	1	Gancao
P74	MOL004904	Licopyranocoumarin	80.36	0.65	1	Gancao
P56	MOL004891	Shinpterocarpin	80.30	0.73	1	Gancao
P82	MOL005017	Phaseol	78.77	0.58	1	Gancao
P211	MOL004841	Licochalcone B	76.76	0.19	1	Gancao
P95	MOL004810	Glyasperin F	75.84	0.54	1	Gancao
P96	MOL001484	Inermine	75.18	0.54	1	Gancao
P200	MOL000500	Vestitol	74.66	0.21	1	Gancao
P80	MOL005007	Glyasperins M	72.67	0.59	1	Gancao
P215	MOL004941	(2R)-7-hydroxy-2-(4-hydroxyphenyl)chroman-4-one	71.12	0.18	1	Gancao
P75	MOL004959	1-Methoxyphaseollidin	69.98	0.64	1	Gancao
P122	MOL004863	3-(3,4-dihydroxyphenyl)-5,7-dihydroxy-8-(3-methylbut-2-enyl)chromone	66.37	0.41	1	Gancao
P54	MOL004903	Liquiritin	65.69	0.74	1	Gancao
P112	MOL004808	Glyasperin B	65.22	0.44	1	Gancao
P152	MOL004829	Glepidotin B	64.46	0.34	1	Gancao
P106	MOL004855	Licoricone	63.58	0.47	1	Gancao
P212	MOL004835	Glypallichalcone	61.60	0.19	1	Gancao
P148	MOL004907	Glyzaglabrin	61.07	0.35	1	Gancao
P135	MOL005000	Gancaonin G	60.44	0.39	1	Gancao
P78	MOL004824	(2S)-6-(2,4-dihydroxyphenyl)-2-(2-hydroxypropan-2-yl)-4-methoxy-2,3-dihydrofuro[3,2-g]chromen-7-one	60.25	0.63	1	Gancao
P115	MOL004849	3-(2,4-dihydroxyphenyl)-8-(1,1-dimethylprop-2-enyl)-7-hydroxy-5-methoxy-coumarin	59.62	0.43	1	Gancao
P83	MOL005003	Licoagrocarpin	58.81	0.58	1	Gancao
P137	MOL004838	8-(6-Hydroxy-2-benzofuranyl)-2,2-dimethyl-5-chromenol	58.44	0.38	1	Gancao
P105	MOL005012	Licoagroisoflavone	57.28	0.49	1	Gancao
P3	MOL005018	Xambioona	54.85	0.87	1	Gancao
P141	MOL005020	Dehydroglyasperins C	53.82	0.37	1	Gancao
P128	MOL004993	8-Prenylated eriodictyol	53.79	0.40	1	Gancao
P107	MOL004908	Glabridin	53.25	0.47	1	Gancao
P161	MOL004910	Glabranin	52.90	0.31	1	Gancao
P108	MOL004879	Glycyrin	52.61	0.47	1	Gancao
P103	MOL004912	Glabrone	52.51	0.50	1	Gancao
P97	MOL004885	Licoisoflavanone	52.47	0.54	1	Gancao
P142	MOL003656	Lupiwighteone	51.64	0.37	1	Gancao
P129	MOL004856	Gancaonin A	51.08	0.40	1	Gancao
P167	MOL000239	Jaranol	50.83	0.29	1	Gancao
P100	MOL004820	Kanzonols W	50.48	0.52	1	Gancao
P30	MOL005001	Gancaonin H	50.10	0.78	1	Gancao
P165	MOL005016	Odoratin	49.95	0.30	1	Gancao
P162	MOL000354	Isorhamnetin	49.60	0.31	1	Gancao, baibiandou
P157	MOL004848	Licochalcone G	49.25	0.32	1	Gancao
P153	MOL002565	Medicarpin	49.22	0.34	1	Gancao
P110	MOL004857	Gancaonin B	48.79	0.45	1	Gancao
P91	MOL004827	Semilicoisoflavone B	48.78	0.55	1	Gancao
P188	MOL000417	Calycosin	47.75	0.24	1	Gancao
P155	MOL004961	Quercetin der.	46.45	0.33	1	Gancao
P163	MOL004898	(E)-3-[3,4-dihydroxy-5-(3-methylbut-2-enyl)phenyl]-1-(2,4-dihydroxyphenyl)prop-2-en-1-one	46.27	0.31	1	Gancao
P113	MOL004911	Glabrene	46.27	0.44	1	Gancao
P85	MOL004974	3′-methoxyglabridin	46.16	0.57	1	Gancao
P130	MOL004811	Glyasperin C	45.56	0.40	1	Gancao
P118	MOL004949	Isolicoflavonol	45.17	0.42	1	Gancao
P149	MOL004828	Glepidotin A	44.72	0.35	1	Gancao
P8	MOL004948	Isoglycyrol	44.70	0.84	1	Gancao
P123	MOL004866	2-(3,4-dihydroxyphenyl)-5,7-dihydroxy-6-(3-methylbut-2-enyl)chromone	44.15	0.41	1	Gancao
P86	MOL004966	3′-hydroxy-4′-O-Methylglabridin	43.71	0.57	1	Gancao
P143	MOL004915	Eurycarpin A	43.28	0.37	1	Gancao
P207	MOL003896	7-Methoxy-2-methyl isoflavone	42.56	0.20	1	Gancao
P119	MOL004883	Licoisoflavone	41.61	0.42	1	Gancao
P79	MOL005008	Glycyrrhiza flavonol A	41.28	0.60	1	Gancao
P1	MOL004924	(-)-Medicocarpin	40.99	0.95	1	Gancao
P156	MOL004980	Inflacoumarin A	39.71	0.33	1	Gancao
P150	MOL004815	(E)-1-(2,4-dihydroxyphenyl)-3-(2,2-dimethylchromen-6-yl)prop-2-en-1-one	39.62	0.35	1	Gancao
P124	MOL004989	6-Prenylated eriodictyol	39.22	0.41	1	Gancao
P92	MOL004884	Licoisoflavone B	38.93	0.55	1	Gancao
P181	MOL004991	7-Acetoxy-2-methylisoflavone	38.92	0.26	1	Gancao
P203	MOL004957	HMO	38.37	0.21	1	Gancao
P158	MOL004945	(2S)-7-hydroxy-2-(4-hydroxyphenyl)-8-(3-methylbut-2-enyl)chroman-4-one	36.57	0.32	1	Gancao
P101	MOL004978	2-[(3R)-8,8-dimethyl-3,4-dihydro-2h-pyrano [6,5-f]chromen-3-yl]-5-methoxyphenol	36.21	0.52	1	Gancao
P125	MOL004935	Sigmoidin-B	34.88	0.41	1	Gancao
P216	MOL001792	DFV	32.76	0.18	1	Gancao
P2	MOL004988	Kanzonol F	32.47	0.89	1	Gancao
P111	MOL004833	Phaseolinisoflavan	32.01	0.45	1	Gancao
P120	MOL004814	Isotrifoliol	31.94	0.42	1	Gancao
P60	MOL004805	(2S)-2-[4-hydroxy-3-(3-methylbut-2-enyl)phenyl]-8,8-dimethyl-2,3-dihydropyrano [2,3-f]chromen-4-one	31.79	0.72	1	Gancao
P126	MOL004864	5,7-Dihydroxy-3-(4-methoxyphenyl)-8-(3-methylbut-2-enyl)chromone	30.49	0.41	1	Gancao
P87	MOL004806	Euchrenone	30.29	0.57	1	Gancao
P210	MOL000230	Pinocembrin	57.56	0.20	1	Gancao
P189	MOL005573	Genkwanin	37.13	0.24	1	Gancao
P176	MOL005575	Gentiacaulein	72.82	0.27	1	Gancao
P147	MOL003673	Wighteone	42.80	0.36	1	Gancao
P177	MOL001735	Hispidulin	30.97	0.27	1	Gancao
P183	MOL003617	Isogosferol	30.07	0.25	1	Gancao
P76	MOL004071	Tetrahydropalmatine	73.94	0.64	1	Gancao
P169	MOL007206	Armepavine	69.31	0.29	1	Lianzi
P144	MOL009172	Pronuciferine	32.75	0.37	1	Lianzi
P131	MOL007213	Nuciferine	34.43	0.40	1	Lianzi
P31	MOL001323	Sitosterol alpha1	43.28	0.78	1	Yiyiren
P213	MOL001494	Mandenol	42.00	0.19	1	Yiyiren
P6	MOL001474	Sanguinarine	37.81	0.86	1	Sharen
P138	MOL006980	Papaverine	64.04	0.38	1	Sharen
P190	MOL000492	(+)-Catechin	54.83	0.24	1	Sharen, baibiandou
P178	MOL004580	Cis-dihydroquercetin	66.44	0.27	1	Jiegeng
P9	MOL008752	Dihydroverticillatine	42.69	0.84	1	Jiegeng
P179	MOL001736	(-)-Taxifolin	60.51	0.27	1	Shanyao
P136	MOL005430	Hancinone C	59.05	0.39	1	Shanyao
P139	MOL000322	Kadsurenone	54.72	0.38	1	Shanyao
P33	MOL005465	AIDS180907	45.33	0.77	1	Shanyao
P180	MOL005267	Elymoclavine	72.87	0.27	1	Shanyao
P214	MOL004058	Deltoside	33.19	0.19	1	Shanyao

**Table 4:** Docking results of target proteins and active compounds.

Target proteins	PDB ID	Compounds	Binding energy (kcal/mol)	Target proteins	PDB ID	Compounds	Binding energy (kcal/mol)
IL6	1ALU	Quercetin	−7.2	AKT1	1UNQ	Quercetin	−6.2
		Luteolin	−7.2			Luteolin	−6.3
		Beta-carotene	−7.6			Beta-carotene	−6.9
		Kaempferol	−6.8			Kaempferol	−6.0
		Naringenin	−6.9			Naringenin	−7.0

ALB	6YG9	Quercetin	−9.8	TP53	5MHC	Quercetin	−7.4
		Luteolin	−10.1			Luteolin	−7.9
		Beta-carotene	−8.2			Beta-carotene	−9.1
		Kaempferol	−8.8			Kaempferol	−7.6
		Naringenin	−8.2			Naringenin	−7.2

VEGFA	1MKK	Quercetin	−7.4	TNF	5UUI	Quercetin	−6.9
		Luteolin	−7.8			Luteolin	−7.0
		Beta-carotene	−7.6			Beta-carotene	−7.3
		Kaempferol	−7.3			Kaempferol	−6.9
		Naringenin	−7.5			Naringenin	−6.4

MAPK3	4QTB	Quercetin	−9.3	CASP3	2DKO	Quercetin	−7.0
		Luteolin	−9.5			Luteolin	−6.9
		Beta-carotene	−9.0			Beta-carotene	−6.2
		Kaempferol	−9.3			Kaempferol	−6.7
		Naringenin	−7.9			Naringenin	−6.5

JUN	6Y3V	Quercetin	−6.5	PTGS2	5F19	Quercetin	−9.7
		Luteolin	−6.5			Luteolin	−10.0
		Beta-carotene	−7.3			Beta-carotene	−8.7
		Kaempferol	−6.3			Kaempferol	−9.3
		Naringenin	−6.5			Naringenin	−8.2

STAT3	6NJS	Quercetin	−8.2	MAPK8	2XRW	Quercetin	−8.2
		Luteolin	−8.0			Luteolin	−8.6
		Beta-carotene	−7.0			Beta-carotene	−9.7
		Kaempferol	−7.9			Kaempferol	−8.6
		Naringenin	−7.2			Naringenin	−6.4

MMP9	6ESM	Quercetin	−10.7	CXCL8	4XDX	Quercetin	−7.5
		Luteolin	−10.9			Luteolin	−7.7
		Beta-carotene	−8.8			Beta-carotene	−9.2
		Kaempferol	−10.3			Kaempferol	−7.6
		Naringenin	−8.7			Naringenin	−6.7

EGFR	5HG8	Quercetin	−8.3	MAPK1	6SLG	Quercetin	−8.1
		Luteolin	−8.6			Luteolin	−8.3
		Beta-carotene	−9.2			Beta-carotene	−8.4
		Kaempferol	−8.5			Kaempferol	−8.1
		Naringenin	−7.9			Naringenin	−7.5

EGF	1JL9	Quercetin	−6.6	MYC	6G6K	Quercetin	−7.2
		Luteolin	−6.8			Luteolin	−7.9
		Beta-carotene	−6.8			Beta-carotene	−7.8
		Kaempferol	−6.8			Kaempferol	−7.6
		Naringenin	−5.9			Naringenin	−7.4

IL1B	5R8Q	Quercetin	−7.1	FOS	1A02	Quercetin	−8.3
		Luteolin	−7.8			Luteolin	−9.3
		Beta-carotene	−7.8			Beta-carotene	−8.8
		Kaempferol	−7.0			Kaempferol	−8.1
		Naringenin	−6.9			Naringenin	−9.3

## Data Availability

The data used and/or analyzed during the current study are available from the corresponding author on reasonable request.
